# Pflügers Archiv - European journal of physiology becomes the official journal of the German Physiological Society

**DOI:** 10.1007/s00424-020-02493-z

**Published:** 2020-11-14

**Authors:** Markus Hecker, Armin Kurtz

**Affiliations:** 1 Institut für Physiologie und Pathophysiologie, Universität Heidelberg, Heidelberg, Germany; 2 Institut für Physiologie, Universität Regensburg, Regensburg, Germany

As the present year comes to an end, the world, for obvious reasons, hopes for a better one to come, with positive developments and promising perspectives. On a much smaller scale, the new year will bring some, as we hope, interesting and welcome changes to this journal. The world as a whole will most certainly be unaware of them, but they will hopefully be registered favorably by the community of physiologists in Germany, Europe and even beyond: Pflügers Archiv - European Journal of Physiology and the German Society of Physiology will team up, and as of January 2021 this publication will be the society’s official journal.

At a first glance it may look strange to have the word “European” in the title of a journal that is about to become the figurehead of a German society. But in fact, it is not. Obviously, the ties between physiological research in Germany and Pflügers Archiv have a long history. In fact, it is the longest one possible since, worldwide, it was the first publication dedicated to this field when it was founded by the German physiologist Eduard Pflüger in 1868. Renamed in his honor in 1910, it is published under its present name since 1968 to reflect the widened scope of nationalities of the researchers who published their work in this journal, but perhaps also to acknowledge the growing importance of international collaborations for the field.

Looking back on close to 120 years of history, the German Physiological Society (DPG) is a youngster by comparison. Today, though, its name is almost more an indication of its linguistic rather than strictly national focus since its membership also includes scientists from Austria and Switzerland. And while its basis lies in this geographic region, the DPG is also firmly rooted in Europe, interacting with its fellow societies, most notably by organizing joint international meetings. So, joining forces with a reputed journal that unmistakably sees itself as an international publication makes perfect sense.

Needless to say, this new partnership will not affect the management and general scope of the journal. *Pflügers Archiv* will continue to have a team of Executive Editors and an Editorial Board consisting of distinguished scientists from all over the world, and it will welcome manuscript submissions from any country and on any area and aspect of physiology, including translational research.

Pflügers Archiv with its editors and the DPG are looking forward to a fruitful collaboration under the roof of the Springer Nature as the publisher, to ensure its success and to shape the future of the journal for, who knows, maybe a further 150 years…

Markus Hecker

President of the German Physiological Society

Armin Kurtz

EiC Pflügers Archiv – Eur J Physiol

